# From Chains to Arrays: Substrate-Mediated Self-Assembly of Diboron Molecules

**DOI:** 10.3390/nano14231952

**Published:** 2024-12-05

**Authors:** Xiaoyu Hao, Huixia Yang, Mengmeng Niu, Tingting Wang, Hongyan Ji, Iulia Emilia Brumboiu, Cesare Grazioli, Ambra Guarnaccio, Albano Cossaro, Yan Li, Jingsi Qiao, Quanzhen Zhang, Liwei Liu, Teng Zhang, Yeliang Wang

**Affiliations:** 1 School of Integrated Circuits and Electronics & Yangtze Delta Region Academy, Beijing Institute of Technology (BIT), Beijing 100081, China; haoxy@bit.edu.cn (X.H.); 3120225780@bit.edu.cn (M.N.); tingting.wang@bit.edu.cn (T.W.); hyji@bit.edu.cn (H.J.); li@bit.edu.cn (Y.L.); qiaojs@bit.edu.cn (J.Q.); quanzhen.zhang@bit.edu.cn (Q.Z.); liwei.liu@bit.edu.cn (L.L.); 2 Faculty of Physics, Astronomy and Informatics, Nicolaus Copernicus University in Toruń, 87-100 Toruń, Poland; iubr@umk.pl; 3 IOM-CNR, Istituto Officina dei Materiali, Basovizza SS-14, Km 163.5, 34149 Trieste, Italy; grazioli@iom.cnr.it (C.G.); acossaro@units.it (A.C.); 4 CNR—Istituto di Struttura della Materia (ISM), 85050 Tito Scalo, Italy; ambra.guarnaccio@pz.ism.cnr.it

**Keywords:** molecular arrays, substrate-mediated self-assembly, epitaxial graphene, 2D materials, organo-boron chemistry, scanning tunneling microscopy

## Abstract

In this study, we explore the substrate-mediated control of self-assembly behavior in diboron molecules (C_12_H_8_B_2_O_4_, B_2_Cat_2_) using scanning tunneling microscopy (STM). The structural transformation of B_2_Cat_2_ molecules from one-dimensional (1D) molecular chains to two-dimensional (2D) molecular arrays was achieved by changing the substrate from Au(111) to bilayer graphene (BLG), highlighting the key role of substrate interactions in determining the assembly structure. Notably, the B-B bond in the molecular arrays on BLG is distinctly pronounced, reflecting a more refined molecular resolution with distinct electronic states than that on Au(111). Density functional theory (DFT) calculations confirm the weak interaction between B_2_Cat_2_ molecules and the BLG substrate, which facilitates the formation of 2D molecular arrays on BLG. This work demonstrates how controlling substrate properties enables the formation of 1D chains and 2D arrays, providing valuable insights for the design of next-generation molecular electronics and catalysis systems.

## 1. Introduction

The ability to actively control molecular self-assembly from one-dimensional (1D) chains to two-dimensional (2D) arrays presents a valuable opportunity to design next-generation nanodevices [1,2,3,4]. In this study, we directly explore different surface arrangements, demonstrating how carefully selecting and fine-tuning substrates can drive the formation of distinct molecular architectures. These different surface arrangements not only improve molecular ordering but also reveal the critical role of substrate interactions in guiding self-assembly, highlighting the importance of deliberate substrate control in determining molecular arrangement [4,5].

The substrate plays a crucial role in influencing molecular self-assembly [6]. Single crystal gold Au(111) and bilayer graphene (BLG) are two substrates with distinct properties, offering unique capabilities for guiding molecular organization. Au(111) is well-known for its herringbone surface reconstruction, where alternating face-centered cubic (fcc) and hexagonal close-packed (hcp) regions modulate the surface free energy and create preferential sites for molecular adsorption [6]. In contrast, BLG, as a representative 2D material, provides a flat, weakly interacting surface, ideal for the formation of ordered molecular arrays [7,8,9,10,11,12]. These contrasting characteristics make Au(111) and BLG effective platforms for investigating how substrate interactions dictate molecular arrangement.

Diboron(4) compounds with B-B bonds hold promise for green and environmental friendly organic-based technologies [13,14,15]. Their stability, reactivity, and synthesis from abundant boron sources align with green chemistry principles, making them ideal for sustainable organic technologies with applications ranging from catalysis to materials science and beyond. Besides its employment in synthesis processes, diboron(4) compounds may be also of potential interest as building blocks in the 2D supramolecular assembly on surfaces composed of boron and oxygen atoms [16]. These 2D supramolecular assemblies are anticipated to exhibit distinctive optoelectronic properties when confined to a 2D surface [17,18,19,20].

Therefore, the bis-catecholato diboron 2-(1,3,2-benzodioxaborol-2-yl)-1,3,2-benzodioxaborole (B_2_Cat_2_) molecule, a simple yet versatile diboron compound, is chosen as model structure for this study. Structurally, B_2_Cat_2_ features a B-B bond within a planar D_2h_ symmetric framework, making it a model system for exploring molecular self-assembly. Despite its structural simplicity, B_2_Cat_2_ has significant potential in materials science, particularly in catalysis, optoelectronics, and molecular electronics [21,22]. The electronic structure of B_2_Cat_2_ has previously been investigated by photoemission (XPS) and near edge X-ray absorption spectroscopy (NEXAFS) experiments [21]. Its electronic structure, including the highest occupied molecular orbital (HOMO) and lowest unoccupied molecular orbital (LUMO), is highly sensitive to substrate interactions, making B_2_Cat_2_ an ideal candidate for studying substrate-driven self-assembly behavior. The combination of its structural simplicity and electronic adaptability makes B_2_Cat_2_ an excellent probe for investigating how substrate interactions control the transition from chains to arrays. However, the specific ordered assembly structure of B_2_Cat_2_ modulated by substrates has not been realized yet.

In this study, we explore how interactions between B_2_Cat_2_ and substrates such as Au(111) and BLG govern the formation of 1D molecular chains and 2D ordered arrays. We use scanning tunneling microscopy (STM) and density functional theory (DFT) calculations to investigate how these substrates precisely modulate molecular interactions and influence the arrangement of B_2_Cat_2_ molecules. The findings provide critical insights into how substrate-induced control of molecular self-assembly can be employed for designing advanced nanomaterials, offering important implications for applications in nanoelectronics and catalysis systems.

## 2. Materials and Methods

Sample preparation and STM measurements. All experiments were carried out under ultrahigh vacuum conditions. The bilayer graphene was epitaxially grown on 6H-SiC(0001) via thermal decomposition at 1200 °C for 40 min [23,24]. The Au(111) single crystal substrate was prepared through repeated cycles of Ar+ ion sputtering (1 keV, grazing incidence) followed by thermal annealing at 700 K to ensure surface cleanliness and reconstruction. B_2_Cat_2_ molecules (Aldrich, 97%) were purified via vacuum sublimation prior to deposition. The purified molecules were then deposited at room temperature (300 K) from a Knudsen-type evaporator onto the substrates (also at room temperature). STM experiments were performed using a commercial scanning tunneling microscope at 5 K with base pressure of 10^−10^ mbar, ensuring high-resolution imaging.

Density functional theory. Density functional theory (DFT) calculations were performed using the generalized gradient approximation (GGA) for the exchange-correlation potential, the projector augmented wave (PAW) method [25]. The calculations used a plane-wave basis set as implemented in the Vienna ab initio simulation package (VASP) [26]. We used five layers of Au(111), which included 160 Au atoms as substrate, and fixed the bottom three layers to simulate the interaction between the B_2_Cat_2_ molecules and Au(111) substrate. For the configuration of B_2_Cat_2_ molecules on BLG, we constructed a 3 × 6 BLG during optimization. We considered both the single-molecule and monolayer cases on BLG and found no significant differences between them. The STM simulation was conducted on a monolayer, while a single molecule was considered for comparison with its behavior on Au. The vacuum layer of each configuration was above 15 Å, and dispersion correction was considered at the DFT-D3 level with the Perdew–Burke–Ernzerhof (PBE) functional [27]. The energy cutoff for the plane wave was set to 400 eV for structural optimization. A k-mesh of 5 × 2 × 1 was adopted to B_2_Cat_2_@Au(111) while B_2_Cat_2_@BLG was 8 × 4 × 1. Total energy convergence criteria were set to 10^−5^ eV, and all atoms were allowed to relax until the residual force per atom was less than 5 × 10^−2^ eV/Å. We calculated DCD by subtracting charge densities of bare substrate (ρ_sub_) and bare B_2_Cat_2_ molecule ($\rho_{B2\mathrm{Cat}2}$) from those in the adsorbed configurations ($\rho_{ads})$, i.e., ρ_DCD_ = $\rho_{ads}-\rho_{sub}-\rho_{B2\mathrm{Cat}2}$. The line profile is the amount of charge in the ab plane integrated along the c-axis.

## 3. Results

**Figure 1:**
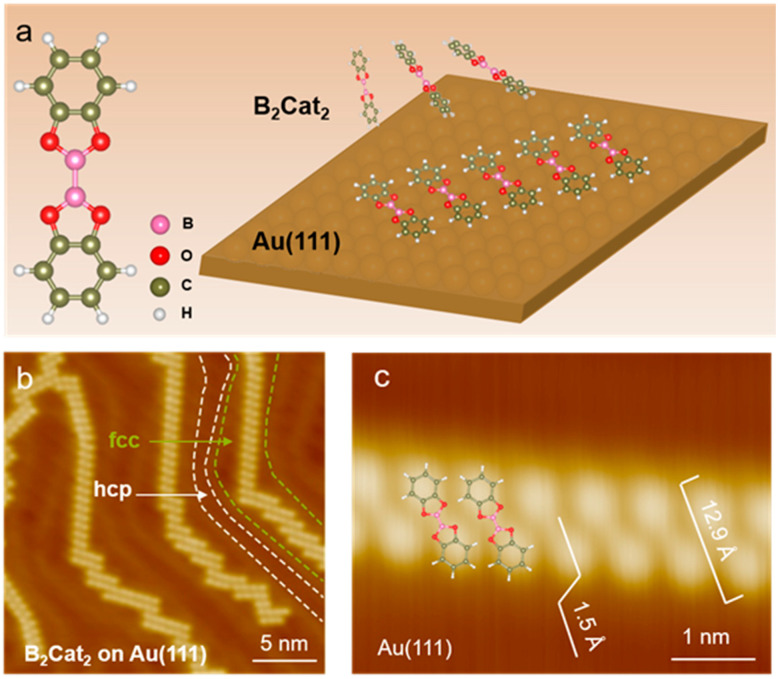
(**a**) (**left**) Schematic of single B_2_Cat_2_ molecule. Boron atoms are depicted in pink, oxygen atoms in red, carbon atoms in green, and hydrogen atoms in white. (**right**) Schematic of B_2_Cat_2_ on Au(111). (**b**) STM image of B_2_Cat_2_ deposited on Au(111) with zoom-in structure presented in (**c**) (parameters: (**b**) V_b_ = −1 V, I_t_ = 5 pA; (**c**) V_b_ = 2.8 V, I_t_ = 100 pA). The length of B_2_Cat_2_ is 12.9 Å, and its height on Au(111) is 1.5 Å.

The B_2_Cat_2_ molecule is planar with D_2h_ point group symmetry. Its molecular structure consists of a segment of a 2D boroxine framework flanked by two phenyl rings (Figure 1a, left panel). The self-assembly of B_2_Cat_2_ molecules on Au(111) and bilayer graphene (BLG) was systematically explored using STM and DFT. Our study demonstrates how substrate properties influence molecular alignment and ordering, leading to a significant difference between one-dimensional (1D) molecular chains on Au(111) and two-dimensional (2D) arrays on BLG.

### 3.1. B_2_Cat_2_ Self-Assembly on Au(111)

As schematically illustrated in the right panel of Figure 1a, we initially investigated the adsorption of B_2_Cat_2_ molecules on Au(111). The Au(111) surface is well known for its herringbone reconstruction, characterized by alternating face-centered cubic (fcc) and hexagonal close-packed (hcp) regions, as marked by dashed lines in Figure 1b [28,29,30]. The fcc regions exhibit higher chemical activity due to their lower local surface density compared to hcp regions [31]. This surface, with its reduced surface free energy and characteristic atomic reconstruction, serves as a common substrate for molecular self-assembly and on-surface reactions. Variations in reactivity and surface interaction energies between the fcc and hcp sites lead to preferential nucleation and reaction sites on the surface.

When deposited on Au(111) (Figure 1b), B_2_Cat_2_ molecules preferentially adsorb on the fcc regions of Au(111), forming 1D zig-zag chains on Au(111) along the elbows of the Au(111) surface. The alignment of these molecular chains along the elbows suggests that the self-assembly structure of B_2_Cat_2_ is heavily influenced by the Au(111) substrate. The formation of these chains along the herringbone direction can be explained by the diffusion barrier, which confines the B_2_Cat_2_ molecules to fcc regions. The STM close-up image in Figure 1b indicates that the B_2_Cat_2_ molecules are parallel to each other and remain confined to the fcc regions, resulting in well-aligned molecular chains. In addition to forming a straight line, the molecule shifts along its major axis to form a polyline configuration (Figure S1). This shift enables the B_2_Cat_2_ molecules to grow along the curved herringbone of the Au(111) surface, forming a limited molecular chain. In studies involving scanning tunneling microscopy (STM) and atomic force microscopy (AFM), molecules have been shown to preferentially adsorb at specific locations on the Au(111) surface [32,33,34]. The Au(111) surface’s role as a template becomes even more apparent in systems like reference [32], where, at higher molecular coverages, the molecules align into preferred orientations within the fcc or hcp regions. This same templating effect is observed in the case of B_2_Cat_2_, where the reconstruction dictates the arrangement of molecules into 1D chains, showcasing the ability of Au(111) to influence both short- and long-range molecular ordering. These studies highlight how the reconstruction of Au(111) is not merely a passive surface but an active participant in molecular self-assembly, directing both the local adsorption behavior and the extended organization of molecules across the surface.

In detail, at the molecular level, each B_2_Cat_2_ molecule presents a central continuous dumbbell-shape appearance. The STM-measured length of a single B_2_Cat_2_ molecule is 12.9 Å with an intermolecular distance 6.8 Å, and the apparent height of the adsorbed B_2_Cat_2_ on Au(111) is 1.5 Å (Figure 1c). As the molecular coverage increases to a full monolayer (Figure S2), the B_2_Cat_2_ chains transform into a disordered, close-packed structure, with no ordered 2D arrangements. The disordered configuration at full monolayer is different from the observed HMBI molecules, which preserve molecular chains when expanded to one monolayer [33]. This disorder can be attributed to the coupling between B_2_Cat_2_ and Au(111), which drives the preferential formation of 1D molecular chains and hinders the development of ordered 2D arrays.

### 3.2. B_2_Cat_2_ Self-Assembly on Bilayer Graphene (BLG)

To synthesize ordered 2D B_2_Cat_2_ molecular structures, we selected a bilayer graphene substrate, a classic atomically thin 2D material with weak interlayer coupling, as a substrate for B_2_Cat_2_ molecules deposition.

**Figure 2:**
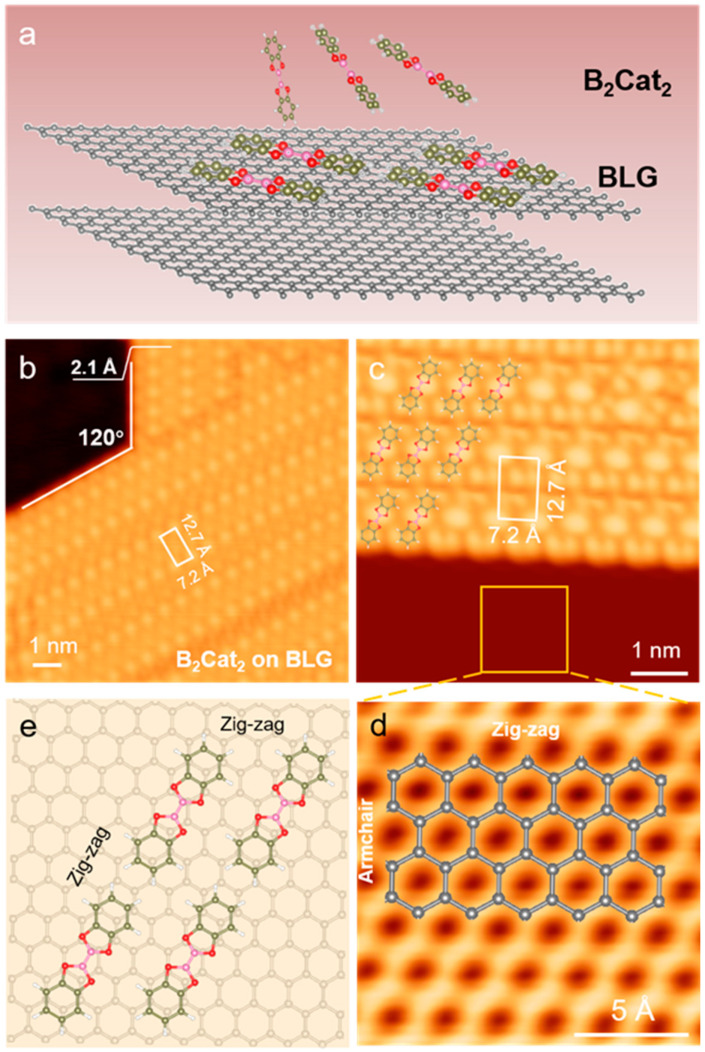
(**a**) Schematic of B_2_Cat_2_ on bilayer graphene (BLG). (**b**) STM image of B_2_Cat_2_ deposited on BLG (V_b_ = −1.5 V, I_t_ = 10 pA). The height of B_2_Cat_2_ on BLG is 2.1 Å. (**c**) Close-up STM image of B_2_Cat_2_ on BLG (V_b_ = −1 V, I_t_ = 50 pA). The white rectangle indicates the unit cell. (**d**) Atomic-resolution STM image of BLG taken from the yellow box in (**c**). (**e**) Schematic description of B_2_Cat_2_ self-assembly structure along zig-zag edge of graphene.

Figure 2a schematically illustrates the deposition process of B_2_Cat_2_ molecules on BLG. The STM image of B_2_Cat_2_ molecules on BLG in Figure 2b shows an array of bright dots with a periodicity of 12.7 Å × 7.2 Å, indicating well-ordered molecular assembly. Additionally, the image reveals the presence of two distinct domains separated by a boundary angle of 120°. The inset line across the domain boundary of B_2_Cat_2_ in Figure 2b indicates that the average height of B_2_Cat_2_ molecules on the BLG is approximately 2.1 Å, confirming that B_2_Cat_2_ molecules are adsorbed further away from the substrate compared to Au(111), suggesting weaker molecule–substrate interactions with graphene.

The close-up STM image (Figure 2c) provides a more detailed view of the self-assembly structure of B_2_Cat_2_ molecules on BLG. In this close-up, individual B_2_Cat_2_ molecules can be clearly distinguished within domains defined by boundaries, as marked by the B_2_Cat_2_ molecular models. The intermolecular distances within the self-assembled structure are measured as 14.7 Å and 7.2 Å, respectively, with an angle of 60° between the molecular rows and columns. This molecular assembly orientation is consistent with the domain boundary angle observed in Figure 2b.

After determining the self-assembled structure of B_2_Cat_2_ molecules, we analyzed the relationship between the molecular arrangement and the BLG substrate. Figure 2d shows a zoomed-in STM image of the BLG region within the yellow box in Figure 2c. By comparing this image with the atomic structure model of BLG (Figure 2d), we confirm that the horizontal direction of the B_2_Cat_2_ molecular array corresponds to the zig-zag edge of the BLG, while the vertical direction corresponds to the armchair edge. Figure 2e shows a schematic representation of B_2_Cat_2_ molecules arranged along the zig-zag edge of the BLG, forming a quadrilateral molecular array.

### 3.3. Electronic Structure Analysis of Molecule–Substrate Interactions

In addition to the difference in molecular assembly structures, STM measurements reveal that B_2_Cat_2_ molecules on BLG exhibit a morphology that is more consistent with their intrinsic electronic structure compared to the dumbbell-shaped appearance observed on Au(111). B_2_Cat_2_ is a kind of diboron molecule with separated electronic states (Figure S2). The highest occupied molecular orbital (HOMO) of B_2_Cat_2_ shows two distinct lobes delocalized over the two benzodioxaborole moieties of B_2_Cat_2_. Instead, the LUMO of B_2_Cat_2_ shows a continuous electronic distribution in the central region of the molecule (Figure S3). The dI/dV spectrum taken from B_2_Cat_2_ molecules reveals HOMO and LUMO energies around −2.2 V and 1.8 V, respectively, defined by the intersections of the tangent at the maximum slope and plateau line (Figure S4) [35].

**Figure 3:**
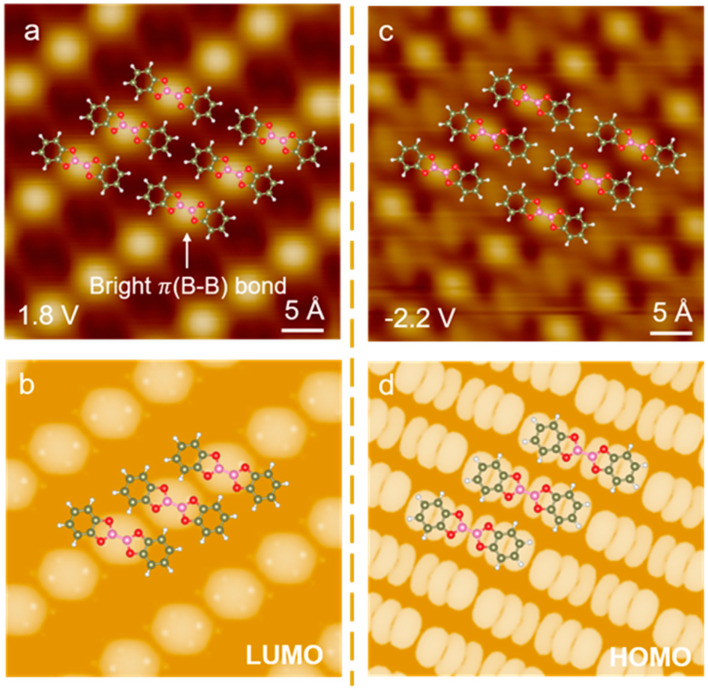
(**a**) STM image of B_2_Cat_2_ molecular array at 1.8 V with tunneling current 10 pA. (**b**) DFT simulation of B_2_Cat_2_ on BLG at LUMO. (**c**) STM image of B_2_Cat_2_ molecular array at −2.2 V with tunneling current 10 pA. (**d**) DFT simulation of B_2_Cat_2_ on BLG at HOMO.

As shown in Figure 3a, the STM image of B_2_Cat_2_ molecules on BLG at 1.8 V exhibits bright dots at the center of B_2_Cat_2_, corresponding to the LUMO which is mainly localized at the molecular center, forming a bright π(B-B) bond, as indicated by the white arrows. STM simulations of B_2_Cat_2_ on BLG at the LUMO state successfully reproduce this localized state at the molecular center (Figure 3b). Similarly, the STM image at −2.2 V (Figure 3c) also presents bright spots at the center of the B_2_Cat_2_ molecules, with additional protrusions observed at both ends of the molecule. The STM simulation at the HOMO state of B_2_Cat_2_ on BLG (Figure 3d) reveals that the electronic states are mainly localized over the two benzodioxaborole moieties, indicating that the protrusions detected at −2.2 V correspond to the HOMO state of the B_2_Cat_2_ molecule. The bright dots observed at −2.2 V in Figure 3c can be attributed to the STM’s working principle, which allows us to measure all the states that are available for tunneling between the Fermi level and the energy corresponding to the applied bias voltage [36].

Overall, the STM images of B_2_Cat_2_ on BLG reveal more detailed structural features compared to those on Au(111). The coupling between the Au(111) substrate and the B_2_Cat_2_ molecules obscures the molecular orbitals, while the weaker coupling with graphene allows for a clearer characterization of the molecular orbital structure. This facilitates a more nuanced detection of the electronic state at the molecular center and over the π state of benzodioxaborole moieties in B_2_Cat_2_.

**Figure 4:**
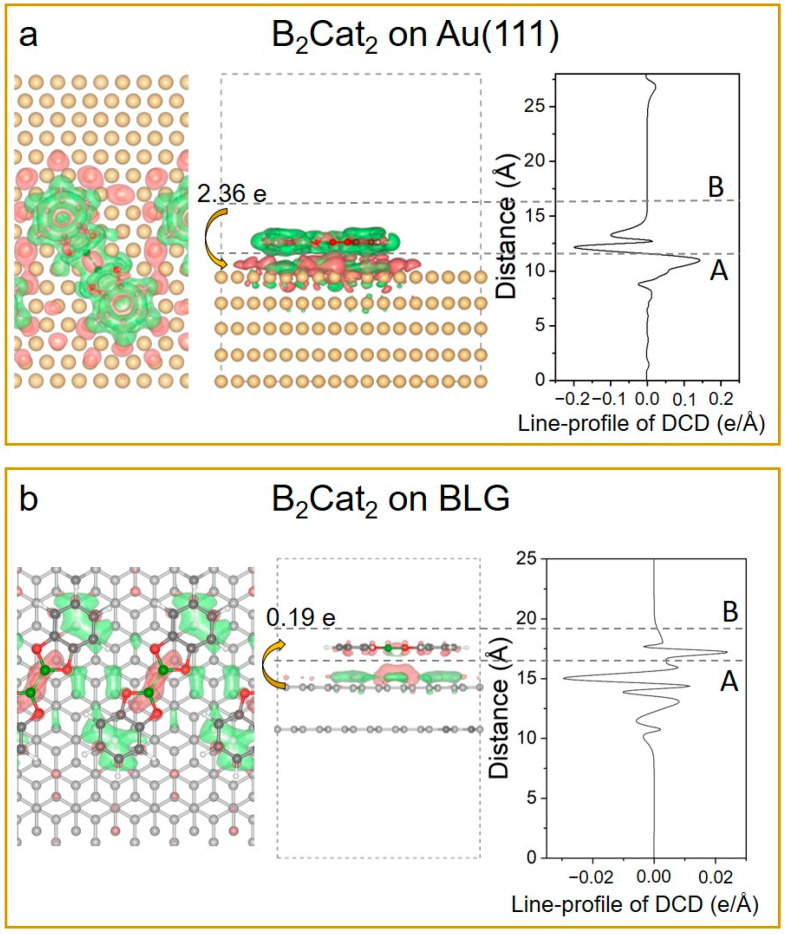
(**a**) Top view (**left**), side view (**middle**), and line-profile (**right**) of differential charge density (DCD) for B_2_Cat_2_ on Au(111). B_2_Cat_2_ donates 2.36 electrons to Au(111), as calculated by integrating line-profile from A to B. (**b**) Top view (**left**), side view (**middle**), and line-profile (**right**) of DCD for B_2_Cat_2_ on BLG. The red (green) regions represent electron accumulation (depletion) with an isosurface value of 2 × 10^−4^ e/Å^3^. B_2_Cat_2_ gains 0.18 electrons from BLG, as calculated by integrating line-profile from A to B.

To gain deeper insight into the formation of 2D molecular arrays on BLG, we performed density functional theory (DFT) calculations to investigate the interactions between the B_2_Cat_2_ molecules and BLG substrate, with Au(111) included for comparison. We constructed models for a B_2_Cat_2_ molecule adsorbed on Au(111) (Figure 4a) and BLG (Figure 4b), respectively. The binding energy of B_2_Cat_2_ on BLG (−1.03 eV) indicates weak coupling compared to its stronger interaction with Au(111) (−2.08 eV). The distance between B_2_Cat_2_ and the BLG was found to be 3.4 Å greater than that between B_2_Cat_2_ and Au(111) (3.2 Å), which further confirms this weaker molecule–substrate interaction in the case of BLG.

It is well established that, as one moves from weaker to stronger interacting substrates, the molecule–substrate distance tends to decrease, often resulting in distortion of the molecular structure. This effect is particularly pronounced on highly interacting substrates such as Cu(111), where significant charge transfer and electronic coupling can occur. However, in the case of Au(111), which is generally considered to be a less interacting surface compared to Cu(111), the extent of molecular distortion is typically minimal.

This observation is supported by the work of Dhum et al., who studied the interaction of PTCDA with different metal substrates (Cu(111), Ag(111), and Au(111)). They found a clear correlation between the electronic properties and the interface geometry, showing that the charge transfer between PTCDA and the substrate decreases as the bonding distance increases along the sequence Cu–Ag–Au [37]. Our findings exhibit a similar trend, where the molecule–substrate distance increases as we transition from Au(111) to BLG, and the associated charge transfer decreases. In fact, in our case, the charge transfer not only decreases but changes sign when moving from Au(111) to BLG, indicating a qualitative change in the interaction mechanism.

Furthermore, the differential charge density (DCD) and corresponding line-profile provide an intuitive and quantitative illustration of the interaction between B_2_Cat_2_ and the substrates, as shown in Figure 4. Figure 4a reveals an obvious electron wave-function distribution, indicating a significant charge transfer between B_2_Cat_2_ and Au(111). The DCD line-profile integral from A to B shows that a B_2_Cat_2_ molecule donates 2.36 electrons to Au(111). In contrast, the line-profile of DCD in Figure 4b demonstrates the weak coupling between B_2_Cat_2_ and BLG, with the B_2_Cat_2_ obtaining only 0.19 electrons from the BLG substrate, significantly lower than the charge transfer observed on Au(111).

This weak coupling facilitates the formation of ordered 2D molecular arrays. It is also noteworthy that the charge distribution in the graphene beneath B_2_Cat_2_ is redistributed, with charge accumulating occurring below the B-B bond. This accumulation likely accounts for the bright spot observed at the center of B_2_Cat_2_ at −2.2 V in Figure 3c. These computational findings demonstrate the effect of substrate interactions on molecular alignment and the visibility of molecular orbitals. The weak interaction between the B_2_Cat_2_ molecules and BLG substrate enables the formation of ordered 2D molecular arrays in a more flexible manner.

An example of a substrate that is sufficiently weakly interacting, allowing the molecular interactions of the deposited molecules to predominantly determine the adlayer morphology, is provided by PTCDA on Ag(111). Ag(111) represents an “intermediate” case between poorly interacting and strongly interacting substrates. In the work of Kilian et al., PTCDA exhibits two distinct adsorption states on the Ag(111) surface: a metastable disordered phase, prepared at low temperatures, and a long-range ordered monolayer phase, obtained at room temperature [38]. These two phases differ significantly in their vertical bonding distances, intramolecular distortions, and electronic structures. The long-range ordered monolayer, in particular, highlights the importance of intermolecular interactions, which dominate the structural arrangement when substrate interactions are relatively weak. This example demonstrates that when the interaction with the substrate is much weaker than the intermolecular forces, the adlayer has greater freedom to reorganize its morphology, emphasizing the need to consider intermolecular forces in such systems.

As a result, by carefully selecting and tailoring the substrate, precise control over molecular self-assembly can be achieved. These findings not only deepen our understanding of substrate–molecule interactions but also provide a theoretical framework for the design and optimization of next-generation organic electronic devices.

## 4. Conclusions

In summary, we successfully demonstrated the self-assembly structural difference of B_2_Cat_2_ molecules from one-dimensional (1D) molecular chains to two-dimensional (2D) arrays, mediated by different substrates. When deposited on Au(111), the B_2_Cat_2_ molecules preferentially form molecular chains along the fcc regions of the surface. However, under full monolayer coverage, a disordered configuration dominates due to the molecule–substrate interactions. In contrast, B_2_Cat_2_ molecules deposited on BLG self-assemble into well-ordered 2D molecular arrays, aligned along the zig-zag edge of BLG, with a more refined molecular structure evident in STM images. The distinct electronic states, particularly the pronounced B-B bond, were clearly visible in these arrays.

Our study elucidates the effect of the substrate on the molecular alignment and ordering. The reconstruction of the Au(111) surface induces the formation of 1D molecular chains of B_2_Cat_2_ molecules on fcc regions at low coverage and hinders the formation of 2D arrays. When B_2_Cat_2_ molecules are deposited on the BLG, the atomic flat surface and weak interaction of the BLG facilitate the self-assembly of B_2_Cat_2_ molecules into ordered 2D arrays.

These findings highlight the potential for controlling molecular self-assembly through substrate selection, particularly in achieving ordered structures on weakly coupled substrates, offering a pathway to designing advanced nanostructures for applications in nanoelectronics, catalysis, and other emerging technologies.

In conclusion, all the cases discussed in this work, including both the systems we studied and the examples provided, pertain to instances of physisorption. As such, these cases involve relatively weak molecule–substrate interactions, where only limited competition with interfacial bonding is expected. Despite this, our findings highlight that the interplay between intermolecular forces and the specific adsorption states observed in our study is of broader relevance for π-conjugated organic molecules on surfaces. This interplay warrants further consideration, both in theoretical calculations of these systems and in the design of novel molecular architectures, as it can significantly influence the structural and electronic properties of the adsorbed layers.

## Data Availability

Data are contained within the article or Supplementary Materials.

## Supplementary Materials

The following supporting information can be downloaded at: https://www.mdpi.com/article/10.3390/nano14231952/s1, Figure S1: STM images of B_2_Cat_2_ chain with dislocation on Au(111); Figure S2: STM images of B_2_Cat_2_ at full monolayer; Figure S3: Calculated frontier molecular orbitals (HOMO and LUMO) of B_2_Cat_2_ in the gas phase; Figure S4: The dI/dV spectra of B_2_Cat_2_ on bilayer graphene; Figure S5: The calculated density of states (DOS) of B_2_Cat_2_ on BLG.
