# Supplementary material for: From Chains to Arrays: Substrate-Mediated Self-Assembly of Diboron Molecules
Source: Nanomaterials (Basel). 2024 Dec 5;14(23):1952. doi: 10.3390/nano14231952 (PMC11643644; doi:10.3390/nano14231952)
Supplement: Supplementary file 1 [file nanomaterials-14-01952-s001.zip › nanomaterials-3301094-supplementary.pdf]

From Chains to Arrays: Substrate-Mediated Self-Assembly of Diboron Molecules

Xiaoyu Hao,[†, #] Mengmeng Niu,[†, #] Tingting Wang,[†] Hongyan Ji,[†] Iulia Emilia Brumboiu,[††] Cesare Grazioli,[‡] Ambra Guarnaccio,[†††] Albano Cossaro,[‡, ††††] Yan Li,[†] Jingsi Qiao,[†] Huixia Yang,[†] Quanzhen Zhang,[†] Liwei Liu,[†] Teng Zhang,[†,] and Yeliang Wang[†,*]*

[†] School of Integrated Circuits and Electronics & Yangtze Delta Region Academy, Beijing Institute of Technology (BIT), Beijing 100081, China.

[‡] IOM-CNR, Istituto Officina dei Materiali, Basovizza SS-14, Km 163.5, 34149 Trieste, Italy.

[††]Faculty of Physics, Astronomy and Informatics, Nicolaus Copernicus University in Toruń, 87-100 Toruń, Poland

[†††]CNR - Istituto di Struttura della Materia (ISM), 85050 Tito Scalo, Italy

†††† Department of Chemical and Pharmaceutical Sciences, University of Trieste, via L. Giorgieri 1, Trieste, Italy

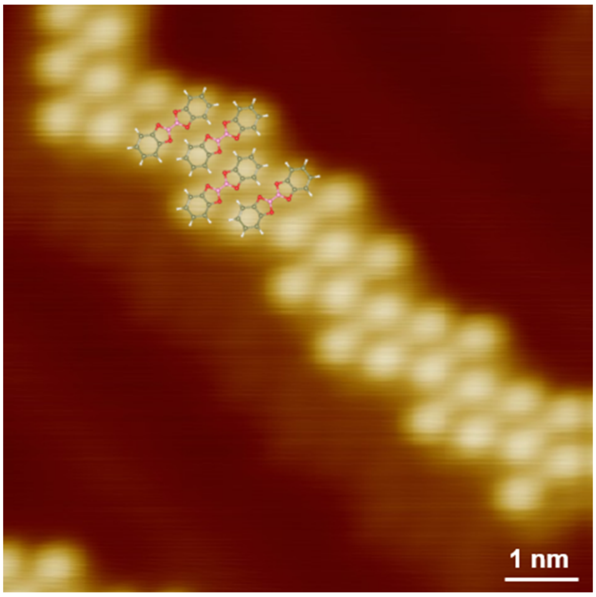

Figure S1. STM images of B_2Cat_2 chain with dislocation on Au(111).

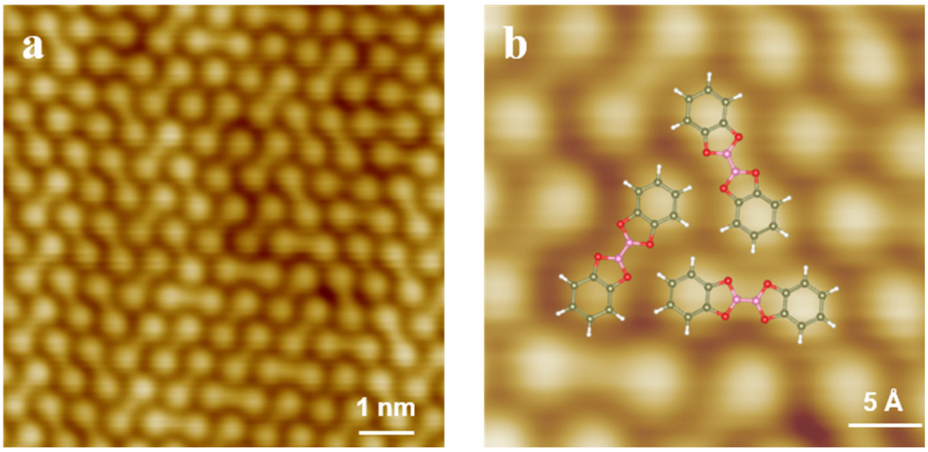

Figure S2. (a) STM images of B_2Cat_2 on Au(111) at full monolayer with zoom-in structure presented in (b).

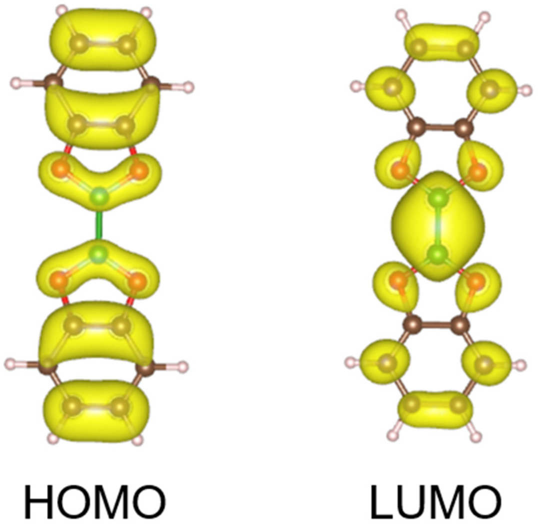

Figure S3. Calculated frontier molecular orbitals (HOMO and LUMO) of B_2Cat_2 in the gas phase.

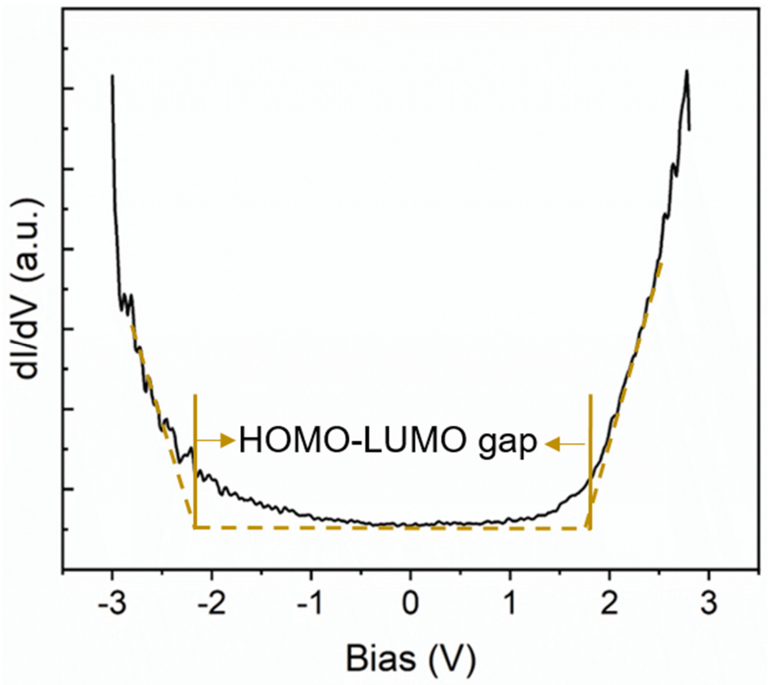

Figure S4. The dI/dV spectra of B_2Cat_2 on bilayer graphene.

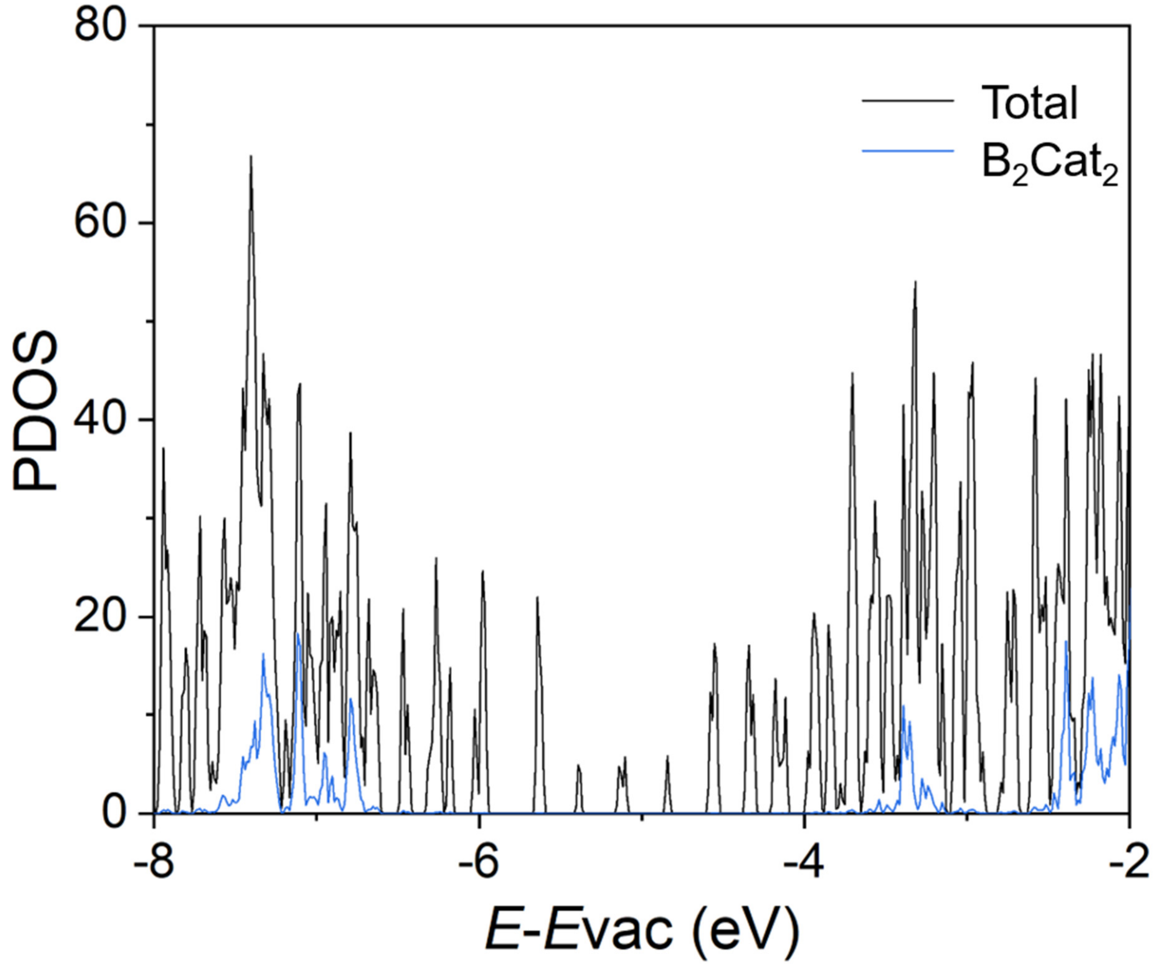

Figure S5. The calculated total density of states (DOS) of B_2Cat_2 on BLG (black lines) and partial density of states (PDOS) of B_2Cat_2 (blue lines).
